# Gut microbiota-mediated immunomodulation in tumor

**DOI:** 10.1186/s13046-021-01983-x

**Published:** 2021-07-03

**Authors:** Xinyi Liu, Yanjie Chen, Si Zhang, Ling Dong

**Affiliations:** 1 Department of Gastroenterology and Hepatology, Zhongshan Hospital, Fudan University, 180 Fenglin Road, Shanghai, 200030 People’s Republic of China; 2 Shanghai Medical College of Fudan University, 130 Dongan Road, Shanghai, 200030 People’s Republic of China; 3 Shanghai Institute of Liver Diseases, Zhongshan Hospital, Fudan University, Shanghai, People’s Republic of China

**Keywords:** Gut microbiota, Tumor immunity, Antibiotics, Immune checkpoint inhibitor

## Abstract

Tumor immunity consists of various types of cells, which serve an important role in antitumor therapy. The gastrointestinal tract is colonized by trillions of microorganisms, which form the gut microbiota. In addition to pathogen defense and maintaining the intestinal ecosystem, gut microbiota also plays a pivotal role in various physiological processes. Recently, the association between these symbionts and cancer, ranging from oncogenesis and cancer progression to resistance or sensitivity to antitumor therapies, has attracted much attention. Metagenome analysis revealed a significant difference between the gut microbial composition of cancer patients and healthy individuals. Moreover, modulation of microbiome could improve therapeutic response to immune checkpoint inhibitors (ICIs). These findings suggest that microbiome is involved in cancer pathogenesis and progression through regulation of tumor immunosurveillance, although the exact mechanisms remain largely unknown. This review focuses on the interaction between the microbiome and tumor immunity, with in-depth discussion regarding the therapeutic potential of modulating gut microbiota in ICIs. Further investigations are warranted before gut microbiota can be introduced into clinical practice.

## Background

**Fig. 1:**
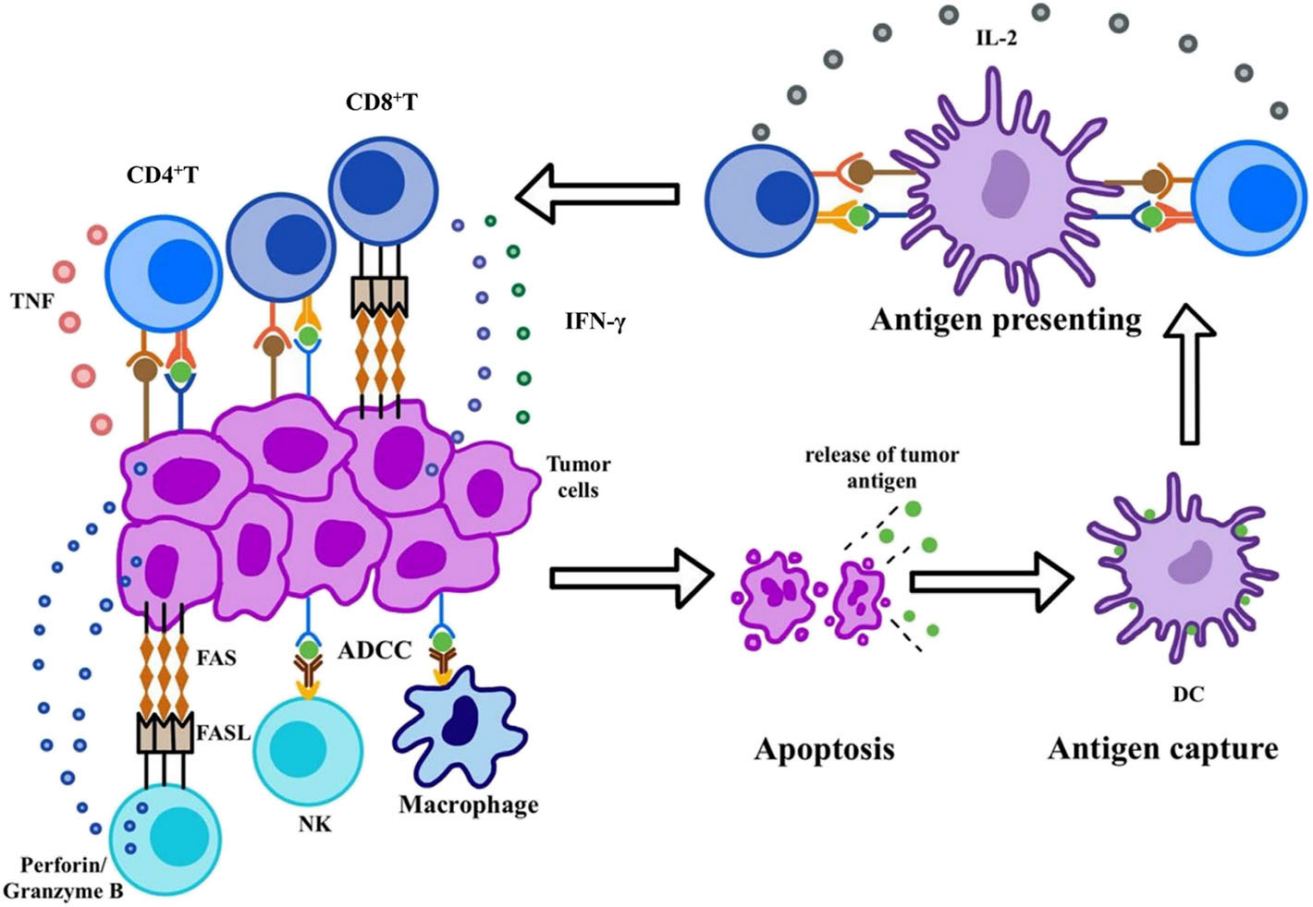
Tumor Immunosurveillance. Tumor immunosurveillance can be divided into two parts, namely innate immunity and adaptive immunity. The former involves various types of myeloid lineage cells and innate lymphoid cells (ILCs), such as macrophage and NK cell. NK cells can kill tumor cells through antibody-dependent cell-mediated cytotoxicity (ADCC), FAS-FASL pathways and perforin-granzyme B. In addition to ADCC and opsonization, macrophages also act as antigen-presenting cells (APC). Adaptive immunity begins with tumor antigen recognized by T cell receptor (TCR), during which dendritic cells (DCs) play a dominant role. Neoantigens generated during oncogenesis are released and captured by DCs for processing. DCs present antigen peptide to T cells in the form of peptide-MHC complex (pMHC). TCR-pMHC interaction combined with costimulatory signal results in the priming of effector T cells. Then the activated T cells, which can specifically target the cancer cells, migrate to the tumor bed and kill the cancer cells through direct cytotoxic effect or producing cytokines to recruit more immune cells

Tumor immunity can be classified as innate immunity or adaptive immunity. Innate immunity involves various types of myeloid lineage cells and innate lymphoid cells (ILCs), including the immune agents they produce [1]. As the first barrier of defense, innate immunity is characterized by its immediate and broad-spectrum response, which is initiated via direct recognition by a limited repertoire of germline-encoded receptors [2]. Conversely, adaptive immunity can execute the target more specifically and accurately. It begins with tumor antigen presentation to T cell receptor (TCR). Neoantigens generated during oncogenesis can undergo presentation by either tumor cells or antigen-presenting cells (APCs), especially dendritic cells (DCs) [3]. Processed antigen peptide is presented to TCR in the form of a peptide-major histocompatibility complex (pMHC) (Fig. 1). TCR-pMHC interaction combined with costimulatory signal leads to the priming of effector T cells (Fig. 1). Then the activated T cells, which can specifically target cancer cells, migrate to the tumor bed and kill the cancer cells through direct cytotoxic effect or producing cytokines to recruit more immunocytes (Fig. 1). Besides, B cells also play a role in antitumor immunity through acting as APCs and secreting cytokines and antibodies. The latter is required for antibody-dependent cell-mediated cytotoxicity (ADCC) mediated by natural killer (NK) cells and macrophages (Fig. 1). However, immunosurveillance against tumor cells is not as effective as expected. Tumor cells can escape immune elimination and even induce immune tolerance through multiple mechanisms such as attenuating antigenicity to disguise as normal cells, down-regulating the expression of MHC I and costimulatory molecules. Moreover, they may even release immunosuppressive cytokines and induce regular T cells (Tregs) and myeloid-derived suppressor cells (MDSCs). Hence, clinical response to cancer immunotherapy varies greatly among individuals. Underlying reasons behind different efficacies have not yet been elucidated. Among many hypotheses, gut microbiota has gradually emerged into public sight.

The gastrointestinal tract is colonized by trillions of microorganisms, which form the gut microbiota. A healthy microbial community plays a pivotal role in many physiological processes, such as pathogen defense, nutrition, metabolism, and immunity [4]. There is growing evidence that compositional and functional alteration of the gut microbiota, referred to as dysbiosis, may be implicated in the pathogenesis of diseases, such as *Clostridium difficile* infection, chronic liver disease, allergy, and metabolic syndrome. Fecal microbiota transplantation (FMT), a way to reconstruct the gut microbiome, has been proved to be a promising therapeutic intervention [5, 6]. Recently, the role of these symbionts in cancer has attracted much attention. Metagenome analysis revealed a significant difference between the gut microbial composition in cancer patients and healthy individuals. For example, patients with colorectal cancer (CRC) have decreased microbial diversity and increased carriage of *Fusobacterium nucleatum*, which is a common resident of the human oral microbiome, but is rarely found in a healthy gut [7, 8]. A high abundance of *F.nucleatum* is also associated with regional lymph node metastasis and shorter survival [9, 10]. Moreover, patients with hepatocellular carcinoma (HCC) showed increased *Bacteroides* and Ruminococcaceae, as well as a lower abundance of *Bifidobacterium* [11]. Although increasing data suggest that gut microbiota is involved in cancer pathogenesis, the underlying mechanisms remain largely unknown. In this review, we focus on the interaction between the gut microbiome and tumor immunity, in attempt to decipher how the commensal microbiome exerts an effect on tumor initiation and progression. We further outlined several important findings in modulating the gut microbiota to enhance the efficacy of immune checkpoint inhibitors (ICIs).

## Potential roles of microbiota in tumor immunity

In the past decade, substantial work has confirmed the role of microbiota in immunity. Kawahara et al. showed that oral administration with *Bifidobacterium longum* can exert anti-influenza virus effect in mice through inducing an increase in NK cell activities and gene expression of IFN-γ, IL-2, IL-12 and IL-18 in the lungs [12]. Even in non-infected mice, probiotic administration also induced significant enhancement in both IFN-γ production and splenetic NK cell activity [12]. Recent evidence demonstrated that microbiome can also influence antitumor response, which may provide a new perspective on improving the efficacy of cancer immunotherapy.

### Innate immunity

#### Macrophage

As an essential component of innate immunity, macrophages have diverse capacities such as direct phagocytosis and cytotoxicity, antigen presentation and immunomodulation. However, it is increasingly appreciated that macrophages in the tumor microenvironment (TME) displayed limited ability to induce antitumor immunity and even function as immunosuppressive cells [13]. Peripheral monocytes recruited to the tumor bed can polarize toward different phenotypes in response to stimuli from TME [14]. They are collectively termed tumor-associated macrophages (TAM), which are classified into M1 (anti-tumor) and M2 (pro-tumor) dichotomy. In tumor initiation, TAM mainly exerts tumoricidal activity, once the tumor has been established, the cells tend to display an M2-like phenotype under the influence of TME [15]. Emerging evidence demonstrated that the disruption of microbiota resulted in immunosuppression via inducing M2-like TAM. Li et al. suggested that antibiotics-induced dysbacteriosis facilitated IL-25-induced activation of M2 macrophages, which promoted HCC progression via secreting CXCL10 and enhancing epithelial mesenchymal transition (EMT) [16]. Further investigation found that dysbacteriosis resulted in hyperplasia of intestinal epithelial tuft cells, from which IL-25 was derived [16]. Another published study identified cathepsin K (CTSK) as a core mediator between dysbacteriosis and malignant progression [17]. Li et al. found that intestinal dysbiosis increased the release of lipopolysaccharide (LPS), which contributed to the expression of CTSK in CRC cells [17]. Overexpression of CTSK was associated with aggressive phenotypes of CRC cells as well as poor prognosis in patients [17]. Further investigation showed that CRC-secreted CTSK activated mTOR pathway via interaction with Toll-like receptor 4 (TLR4) on the macrophage membrane, inducing M2 polarization and production of cytokines, such as IL-4, IL-10 [17]. Hence, conversion from M2 to M1 macrophages may be a promising target in cancer immunotherapy. A favorable microbiome can likely facilitate this re-polarization. An in-vitro experiment found that *Bacteroides fragilis* promoted phagocytosis of macrophages and their polarization towards M1 phenotype [18].

#### MDSC

Normally, bone marrow-derived immature myeloid cells (IMC) differentiate into macrophages, neutrophils and DC [19]. In the presence of chronic inflammation like the one mediated by cancer, the differentiation is impaired, leading to the accumulation of IMC with immunosuppressive functions, namely MDSC [20]. They contribute to an immunosuppressive TME via multiple mechanisms, which have been described in-detail in a recent review [19]. Some gut microbiota were reported to contribute to oncogenesis and tumor progression in an MDSC-dependent manner. For instance, colonization of mice with Enterotoxigenic *Bacteroides fragilis* was confirmed to trigger Th17-dependent recruitment of myeloid cells to the TME as well as their differentiation towards immunosuppressive MDSCs, which promoted colon tumorigenesis [21].

#### Other innate Immunocytes

In addition to the above-mentioned cells, other innate immunocytes are also involved in tumor cytotoxicity. Recruited neutrophils can exert antitumoral activity via production of reactive oxygen species (ROS). NK cells can target tumor cells, which down-regulate the expression of MHC-I to escape attack from cytotoxic T lymphocytes [22, 23]. γδT cells express γδ T cell receptor and do not require antigen presentation from APCs, which make them react earlier than conventional αβT cells [24, 25]. It is reported that gut microbiota can also influence these cells. Iida et al. found that the reduced effect of oxaliplatin in germ-free mice is partially due to reduced ROS production from neutrophils [26]. *Barnesiella intestinihominis* was reported to exert an adjuvant impact on cyclophosphamide (CTX)-induced tumor immunity by promoting infiltration of IFN-γ-producing γδT cells in cancer lesions [27]. These findings indicate that a favorable microbiome is required for normal functions of tumor-infiltrating leukocytes and a better therapeutic response. Gur et al. found that Fap2 protein of *Fusobacterium nucleatum* could specifically target the inhibitory receptor TIGIT, which is present on human NK cells, and therefore inhibits the cytotoxic effect [9]. A clinical study in patients with non-small-cell lung cancer (NSCLC) indicated that a higher diversity of microbiome was correlated with greater frequencies of peripheral NK cells and a better response to ICIs [28]. Conversely, antibiotics treatment altered the intestinal microbiota at a family level, followed by reduced cytotoxic NK cells and increased growth of intracranial glioma, suggesting that an abundant microbiome facilitated the accumulation of NK cells and enhanced tumor surveillance [29].

### Adaptive immunity

#### Dendritic cell

Although DC belongs to innate immunity, it is discussed in adaptive immunity, considering its critical role in initiating T cell-mediated immune response [30]. Similar to macrophages, TME-mediated immunosuppression can induce dysfunction of DC, leading to failure in T cell priming [30]. Recent data have revealed that some gut commensals may enhance immune response by regulating DC, providing a new perspective on improving efficacy of immunotherapy. Both *Bacteroides fragilis* [31] and *Bifidobacterium* [32] were reported to promote the activation and maturation of DCs. An abundance of Ruminococcaceae was associated with a higher expression of markers of antigen processing and presentation [33]. Apart from the role of antigen presentation, DCs also provide co-stimulatory signals for T cell activation [34]. Iida et al. found that antibiotics decreased CD86 (B7–2) expression in tumor-associated DC [26]. As the ligand of CD28 on T cells, the combination contributes to the expression and production of IL-2, facilitating the proliferation and differentiation of T cells.

#### Effector T cell

On one hand, DCs cross-present tumor antigens on MHC-I molecule to CD8^+^T cells and induce them to differentiate into cytotoxic T lymphocytes (CTLs). CTLs can not only utilize perforin-granzyme pathway and death ligand to mediate tumor cell apoptosis, but can also secret a series of cytokines, such as IFN-γ and TNF-α, to exert direct cytotoxicity or interact with other immune cells. On the other hand, CD4^+^T cells are activated by endogenous antigens presented on MHC-II molecule and then differentiate into helper T cells (Th). CD4^+^Th cells can create a positive immune environment and facilitate the accumulation and activation of other immunocytes in a cytokine-dependent manner. Due to the dominant role of T cells in tumor surveillance, most immunotherapies focus on increasing tumor-infiltrating T cells or releasing them from repression by TME. Microbiome was also discovered to prime T cells for tumor cytotoxicity. Tanoue et al. isolated 11 strains of fecal microbiota from healthy volunteers and found that the bacterial mixture was capable of inducing IFNγ^+^CD8^+^T cells in recipient mice, exerting an independent antitumor effect [35]. Analysis of fecal samples revealed that the enrichment of specific gut commensals, such as *Bifidobacterium* [32] and Ruminococcaceae [33], had a significant positive correlation with CD8^+^T cell infiltration in the tumor bed or tumor-draining lymph node. Furthermore, gut commensals also stimulate effector T cells via cytokine production. As an activator of Th1 response, IFN-γ could not only exert direct cytotoxic effects and upregulate MHC-I in tumor cells, but also modulate the expression of perforin and granzyme. *Bifidobacterium*-treated mice showed increased IFN-γ production, followed by stronger tumor-specific T cell responses and slower tumor growth [36]. In contrast, antibiotics-induced dysbiosis promoted tumor growth via a suppressed level of TNF-α and a subsequent decrease in tumor endothelial adhesion molecules, especially intercellular adhesion molecule 1 (ICAM-1). The latter plays a crucial role in the trafficking of leukocytes into tumor tissue [37]. As a consequence, the number of activated CD8^+^T cells decreased [37].

#### Regulatory T cell

As an immunosuppressive subset of CD4^+^T cells, Tregs are characterized by constitutive expression of high-affinity IL-2 receptor but limited secretion of IL-2 [38]. Thus, exogenous IL-2, which mainly derives from activated T cells, is indispensable for their survival and functions [38]. Tregs mediate negative immune response via direct contact with target cells and release of immunosuppressive molecules like TGF-β and IL-10 [38]. Normally, they are indispensable for the maintenance of autoimmune tolerance and immune homeostasis. However, in the setting of neoplasia, they are responsible for immune escape. A large number of data confirmed that an abundance of Tregs in TME predicted poor prognosis in patients [39, 40]. Thus, targeting Tregs to reverse suppressive TME may be an effective strategy in cancer immunotherapy. Recent studies showed that patients whose baseline microbiota was driven by *Faecalibacterium* genus and other Firmicutes had a lower proportion of peripheral Tregs [41], while a fecal microbiome rich in Bacteroidales was correlated with a higher level of Tregs [33]. Furthermore, germ-free mice receiving FMT with a high abundance of Bacteroidales also showed a higher level of CD4^+^Foxp3^+^T cells in the spleen [33], insinuating the reduction of Tregs via colonization with a favorable microbiome.

#### B cell

B cells participate in tumor surveillance by secreting immunoglobulins and cytokines, as well as serving as APCs. But every coin has two sides. Among various subtypes, regulatory B cells can suppress antitumor immunity via the secretion of immunosuppressive cytokines, such as IL-10 and TGF-β, and the induction of Tregs [42, 43]. Previous findings have revealed that gut commensals can tightly interact with B cells. Gut microbiota-derived antigens bind to various receptors on B cells to mediate B cell activation and differentiation [44]. They can also exert an indirect effect on B cells through epithelial cells, T cells, and myeloid cells [44]. Besides, commensal microbiota is required for normal levels of IgA, which serves as an essential part of mucosal immunity [45]. In turn, defective IgA secretion or function induces microbial dysbiosis [46, 47]. Moreover, it was shown that resident microbiota also stimulated the regulatory capacity of B cells to reduce colonic T cell activation, maintaining mucosal homeostasis [48]. Ramakrishna et al. demonstrated that *Bacteroides fragilis* or its capsular polysaccharide A could bind to enteric B cells to induce IL-10 production and restrain innate inflammatory responses in the central nervous system [49]. However, evidence about microbiota-modulated B cells in tumor immunity remains scarce, which requires more attention in future studies.

### Heterogeneity of Immunocytes and dual function of inflammation

With regards to the impact of microbiota on immunocytes, it is important to note that tumor-infiltrating immunocytes show great plasticity in terms of subsets and functions. Hence, it is inappropriate to simply define their immunological effects as tumor-promoting or tumor-suppressing. For example, Foxp3^+^Treg is not absolutely related to immunosuppression. Miyara et al. divided CD4^+^Foxp3^+^Treg cells into three subpopulations by combining Foxp3 and CD45RA staining: 1) FoxP3^lo^CD45RA^+^cells: resting Tregs which proliferated and converted into activated Tregs; 2) FoxP3^hi^CD45RA^−^cells: activated Tregs which was terminally differentiated and highly suppressive; 3) FoxP3^lo^CD45RA^−^cells: non-suppressive Tregs which produced large amounts of IL-2 and IFN-γ [50]. A recent study further supported the existence of heterogenous Foxp3^+^Treg in CRC, which correlated with different prognosis [51]. Tregs with low expression of FOXP3 exhibited markedly lower expression of immunosuppressive molecules, indicating better prognosis in CRC patients, compared to Tregs with high expression of FOXP3 [51]. Hence, assessing the immunological effect of gut microbiota-induced Tregs without functional and phenotypic analysis may cause contradictory results. Likewise, it is acknowledged that γδT cells exhibit an immunosuppressive phenotype via IL-17 production [52]. IL-17-producing γδT cells (γδT17) in TME was associated with higher relapse, lymph node metastasis and increased mortality rates [53]. However, a beneficial role for γδT17 in microbiota-mediated tumor regression was also reported. Cheng et al. found that antibiotics-treated mice exhibited a defective induction of γδT17 cell response, leading to more and larger tumor foci as well as a shorter survival time [54]. Adding γδT cells or supplementing IL-17 could restore the impaired immune surveillance in antibiotics-treated mice [54].

Additionally, even though immunocytes and cytokines are key components in tumor surveillance, they may also contribute to tumor progression by modulating inflammation [55]. For instance, activation of CD8^+^T cells and NF-κB signaling could facilitate non-alcoholic steatohepatitis (NASH)-to-HCC transition [56]. Conversely, preventive probiotic feeding could significantly inhibit HCC growth in mice by modulation of gut microbiota, which promoted the differentiation of anti-inflammatory Treg/Tr1 cells in the gut and reduced the recruitment of proinflammatory Th17 to the liver [57]. The immunological effects may be more complicated when considering different tumor types and staging. Hence, more evidence is expected before harnessing microbiome in cancer therapy.

Taken together, the immune system is an integration of various immune cells and cytokines. The immunological functions of microbiome may be synergistic between innate and adaptive immunity, since targeting a single cell or molecule has a limited effect. Thus, further investigations are needed to identify species that can activate multiple immunocytes to amplify antitumor response.

### Potential mechanisms for microbiota-mediated immunomodulation in tumor

#### PAMP-TLR/NF-κB interaction

**Fig. 2:**
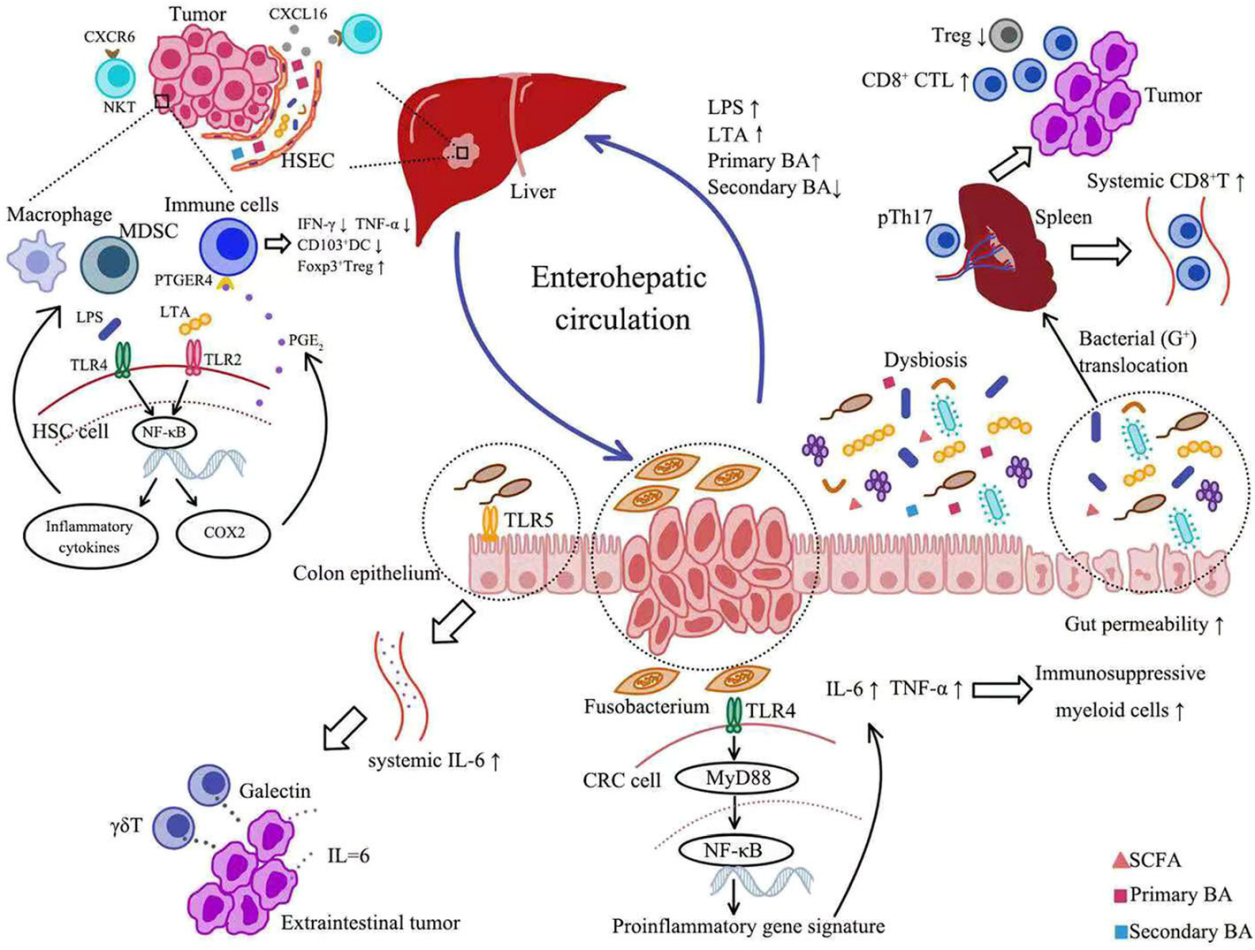
Potential Mechanisms for Microbiota-Mediated Immunomodulation in Tumor (see attached file). Gut microflora can exert an impact on tumor immunity both locally and systemically. Locally, Fusobacterium may act on CRC cells via TLR4/MYD88 signaling pathway. The activation of NF-kB promoted the transcription of pro-inflammatory cytokines such as TNF-α and IL-6, leading to the accumulation of immunosuppressive myeloid cells in TME. Systemically, bacterial flagellin accelerated distal malignant progression via TLR5 signaling, resulting in increased systemic IL-6 and subsequent more γδT cells to produce immunosuppressive galectin-1. Furthermore, the enterohepatic circulation enabled microbiota-derived PAMP and metabolites to play a role in HCC. On one hand, in the context of HCC, there is a significant increase in portal and systemic LPS, owing to dysbiosis and increased gut permeability. Elevated LPS activated NF-κB in HSC, inducing production of inflammatory chemokines. These cytokines could enhance migration of macrophages and MDSCs to the liver. Similarly, gut-derived LTA induced the expression of COX2 to promote local production of PGE_2._ Then PGE_2_ suppressed the antitumor response through the PTGER4 receptor on immune cells, manifested as decreased production of IFN-γ and TNF-α, reduced CD103^+^DC and increased CD4^+^FOXP3^+^Treg. On the other hand, depletion of gram-positive bacteria involved in primary-to-secondary bile acid conversion increased the expression of CXCL16. Upregulation of CXCL16 induced accumulation and activation of CXCR6^+^NKT cells, which suppressed liver tumor growth. In addition, intestinal microbiota could also control the immune tone of secondary lymphoid organs via bacterial translocation. The translocation of selected Gram-positive bacterial species into spleen is indispensable for CTX-driven accumulation of pTh17 cells, which increased systemic CD8^+^T cells and intratumoral CTL/Treg ratio. BA: bile acid; CRC: colorectal cancer; TLR: Toll-like receptor; TME: tumor microenvironment; PAMP: pathogen-associated molecule pattern; HCC: hepatocellular carcinoma; LPS: lipopolysaccharide; MDSC: myeloid-derived suppressor cells; LTA: lipoteichoic acid; HSC: hepatic stellate cell; HSEC: hepatic sinusoidal endothelial cell; SCFA: short-chain fatty acids

Aforementioned findings suggested that microbiota could influence tumor immunity via interaction with various immunocytes, but the underlying mechanisms remain elusive. It is well established that innate immunity is triggered upon recognition of pathogen-associated molecule pattern (PAMP) by pattern recognition receptor (PRR). Previous study has shown that cell surface polysaccharides of *Bifidobacterium bifidum* could activate Toll-like receptor 2 (TLR2)/MyD88 pathway to induce Tregs, displaying robust suppressive capacity toward experimental colitis [58]. Hence, it is reasonable that microbiota-derived PAMP can act on PRR, which regulates immune response against tumor. Among various pathways, TLR/MyD88/nuclear factor-κB (NF-κB) signaling is the most well-known. Kostic et al. found a correlation between a high abundance of *F. nucleatum* and activated NF-κB in CRC. The activation of NF-kB promoted the transcription of pro-inflammatory cytokines such as TNF-α and IL-6, which may explain the accumulation of immunosuppressive myeloid cells in TME [59] (Fig. 2). The notion was further supported by another study showing bacterial flagellin stimulated pro-tumoral inflammation through TLR5 signaling [60]. The interaction resulted in IL-6-dependent mobilization of MDSCs and subsequently more γδT cells producing immunosuppressive galectin-1, followed by impaired antitumor response and accelerated malignant progression [60] (Fig. 2).

Of note, PAMP-mediated immunomodulation also makes sense in tumors outside the digestive tract. Among various tumor types, HCC is the most well-studied since the liver is intricately linked with the intestinal tract both anatomically and functionally, namely the gut-liver axis [61]. Hence, gut microbiota and their metabolites can exert an effect on liver cancer via the enterohepatic circulation. Ren et al. found that LPS-producing bacteria were enriched in patients with early HCC versus controls, suggesting a potential role for LPS in HCC development [62]. LPS, a specific component of gram-negative bacterial cell wall, triggers innate immunity through interaction with TLR4. Normally, LPS transported to liver through the portal system is rapidly cleared by Kupffer cells [63]. However, in the context of chronic liver diseases, there is a significant increase in portal and systemic LPS, owing to dysbiosis and increased gut permeability [61] (Fig. 2). Accumulating data demonstrated that gut-derived LPS induced activation of hepatic stellate cells (HSC) to drive fibrogenesis and subsequent malignant transformation. Elevated LPS activated NF-κB in HSC via TLR-4 signaling, followed by production of inflammatory chemokines and expression of cell surface adhesion molecules [63, 64] (Fig. 2). These cytokines could enhance migration of macrophages and MDSCs to the liver [64, 65] (Fig. 2). Furthermore, LPS sensitized HSC to TGF-β-mediating activation in a TLR4/MyD88/NF-κB-dependent manner [64]. The activated HSCs were reported to preferentially affect monocyte populations and shift their gene expression from an inflammatory to an immunosuppressive signature, supporting HCC development [66]. However, LPS can also function as a key component in activation of tumor immunity. B16 melanoma-bearing mice showed a diminished TNF production by tumor-associated myeloid cells after antibiotics treatment and thus responded poorly to immunotherapy. Oral gavage with LPS could largely restore the amount of intratumoral TNF-producing leukocytes and therapeutic response in wild-type, but not TLR4-deficiency mice, suggesting LPS-mediated TLR4 activation was required for the immunological effect of microbiota [26]. However, how intestinal flora-derived PAMPs affect cytokine production in other distant organs requires further investigation.

In addition to LPS, lipoteichoic acid (LTA), the major cell wall component of Gram-positive bacteria, also participated in immunosuppression in obesity-associated liver cancer [67]. Loo et al. found that mice with obesity-associated liver cancer exhibited a dramatic increase in Gram-positive gut microbiota, accompanied by increased LTA in the liver tumor tissues [67, 68]. LTA induced the expression of cyclooxyganese2 (COX2) to promote local production of PGE2 in senescent HSCs through TLR2-NF-κB signaling [67] (Fig. 2). Then PGE2 suppressed the antitumor response through the PTGER4 receptor on immune cells, which manifested as decreased production of IFN-γ and TNF-α, reduced CD103^+^DC and increased CD4^+^FOXP3^+^Tregs [67] (Fig. 2).

Collectively, these findings suggested a potential role for TLR-PAMP combination in microbiota-mediated antitumor response. In this sense, genetic polymorphisms of PRR can hamper the interactions between the microbiome and immune system, which may account for different sensitivity to microbiota-targeted therapy in patients [69]. Of note, given that PAMPs, such as LPS and flagellin, are widely expressed in a group of bacteria, these findings imply that all the gut commensals with the same PAMPs possess similar immunological effects. It is inconsistent with previous studies, indicating more mechanisms to explain the immunomodulation induced by gut microbiota.

#### Microbiota-derived metabolites

Considering the vital role of microbiota in metabolism, it is reasonable to assume that their metabolites are implicated in the regulation of tumor immunity. Analysis of fecal samples from early HCC patients displayed a decrease in butyrate-producing bacteria, indicating that short-chain fatty acids (SCFAs) are associated with HCC development [62]. SCFAs are microbial fermentation products produced in the colon. It is increasingly appreciated that SCFAs, in particular propionate and butyrate, mediate anti-inflammatory and immunosuppressive effects via interaction with G protein-coupled receptor (GPR) [70–72]. Singh et al. demonstrated that butyrate was an agonist for GPR109A [73]. Gpr109a signaling promoted anti-inflammatory properties in colonic macrophages and dendritic cells and enabled them to induce differentiation of naïve T cells into Treg cells [73]. Gpr109a-deficiency mice were susceptible to colitis and inflammation-induced colon carcinogenesis [73]. Likewise, propionate-induced anti-inflammatory effects were dependent on GPR 43 [74]. In addition, both propionate and butyrate could act as histone deacetylase (HDAC) inhibitor [74–76], through which SCFA increased histone acetylation in the promoter and conserved non-coding sequence (CNS) regions of the Foxp3 locus, the master transcription factor and specific marker of suppressive Tregs [75]. These findings highlighted the immunosuppressive role of SCFA. However, Kespohl et al. described that butyrate could exert bidirectional effects, depending on its concentration and immunological milieu [77]. In line with previous studies, lower concentration of butyrate facilitated differentiation of Tregs, whereas high concentration without TGF-β could induce expression of the transcription factor T-bet in all investigated T cell subsets and promote IFN-γ production, contributing to polarization towards Th1 cells [77].

Bile acid is another metabolite attracting much attention in recent years. In humans, cholesterol-derived primary bile acids are mostly conjugated with glycine or taurine before excretion into bile and further passed into the duodenum [78]. Then they undergo further processing performed by gut bacteria, giving rise to secondary bile acids [78]. About 95% of bile acids are reabsorbed via enterohepatic circulation [78]. Previous studies have demonstrated bile acids mediated anti-inflammatory effects via stimulation of receptors TGR5 and farnesoid X receptor (FXR) [79, 80]. Mcmahan et al. found that simultaneous activation of FXR and TGR5 resulted in intrahepatic accumulation of Ly6C^low^ monocytes, which subsequently differentiated into anti-inflammatory macrophages [79]. Moreover, agonism of FXR and TGR5 inhibited production of proinflammatory cytokines by hepatic macrophages, inducing a phenotypic switch to M2-like macrophages [79]. Although the anti-inflammatory effects enable bile acids to attenuate inflammation-associated damage, immunosuppression may also drive carcinogenesis.

Another recent study showed blocking bile acid biotransformation suppressed liver cancer through chemoattracting natural killer T cells (NKT), which are innate-like T lymphocytes expressing both TCR and innate-immune-like receptors. They recognize lipid antigens presented by molecule CD1d [81–83]. Ma et al. found that primary bile acids increased CXCL16 expression, whereas secondary bile acids showed the opposite effect [81]. Antibiotics treatment caused depletion of gram-positive bacteria involved in primary-to-secondary bile acid conversion, therefore increasing the expression of CXCL16 [81] (Fig. 2). As the only ligand for CXCR6, upregulation of CXCL16 induced accumulation and activation of CXCR6^+^NKT cells, which produced more IFN-γ and suppressed liver tumor growth [81] (Fig. 2). Feeding secondary bile acids or colonization with bile acid-metabolizing bacteria abrogated both NKT cell accumulation and tumor inhibition in mice [81]. Similar findings were confirmed in patients with primary liver cancer [81].

#### Immunomodulation in secondary lymphoid organs

Previous studies have confirmed that gut microbiota also influenced neoplasia outside the gastrointestinal tract, indicating an access for gut commensals to exert a systemic effect. Recent studies showed that the intestinal microbiota ecosystem might control not only the gut immune homeostasis but also the immune tone of secondary lymphoid organs via bacterial translocation, ultimately shaping the TME. Viaud et al. found that CTX compromised the integrity of the intestinal barrier, leading to translocation of selected Gram-positive bacterial species (including *Lactobacillus johnsonii* and *Enterococcus hirae*) into secondary lymphoid organs, which is indispensable for CTX-driven accumulation of pTh17 cells (which share hallmarks of Th1 cells and Th17 cells) and therapeutic effects [84] (Fig. 2). Further analysis showed that the translocation of *E. hirae* to secondary lymphoid organs could exert an adjuvant impact on systemic and local immune responses mediated by CTX [2]. Systemically, *E. hirae* facilitated the accumulation of effector CD8^+^T cells [27] (Fig. 2). Locally, it increased the intratumoral CTL/Treg ratio [27] (Fig. 2). Intriguingly, both studies highlighted memory Th1 cells response against specific bacteria following CTX treatment. Moreover, Daillere et al. found that memory Th1 cells recognizing *E. hirae* and *B. intestinihominis* predicted longer progression-free survival (PFS) in advanced lung and ovarian cancer patients treated with chemotherapy [27]. In support of this notion, another study found that circulating CD8^+^T cells from HBV-related HCC patients demonstrated significantly elevated responses to bacteria including *Escherichia coli*, *Enterococcus faecium*, *Bifidobacterium longum*, *Bacteroides fragilis*, and *Enterococcus hirae*, compared to healthy controls [85]. These bacteria-reactive responses depended on the presence of antigen-presenting monocytes and were MHC class I-restricted [85]. Furthermore, they also observed that the proportions of *Bifidobacterium longum*-reactive and *Enterococcus hirae*-reactive CD8^+^T cells were positively correlated with CD8^+^T cell-to-Foxp3^+^Treg ratio, as well as the disease-free survival (DFS) time of HCC patients after tumor resection [85]. These findings confirm a link between bacteria-specific T cell response, enhanced antitumor immunity and better outcomes, suggesting a potential molecular mimicry between specific commensals and tumor antigens [86].

Although the exact mechanisms for microbiota-mediated tumor immunity remain largely unknown, existing evidence suggests a potential cause and effect relationship, in which gut microbiome may have a distinct influence on tumor immunity both locally and systemically. These findings prompt the possibility to harness microbiome in cancer immunotherapy.

## Gut microbiota and ICIs

### Role of immune checkpoint in tumor immunity

Immune checkpoint proteins, including cytotoxic T-lymphocyte antigen-4 (CTLA-4), programmed death 1 (PD-1) and its ligand programmed death ligand 1 (PD-L1), can deliver inhibitory signals to negatively regulate the immune system. PD-1 is mainly expressed on activated T, B and myeloid cells, while its ligand PD-L1 is widely expressed on various immune and non-immune cells [87]. Upon T cell activation, cytokines secreted from activated tumor-infiltrating lymphocytes (TIL), such as IFN-γ, can induce the expression of PD-L1 in TME. Ligation between PD-1 and PD-L1 leads to anergy, exhaustion and apoptosis of activated T cells via inhibiting PI3K-Akt and Ras-MEK-Erk signaling pathways [88, 89]. In addition, accumulating studies demonstrated that PD-1/PD-L1 axis also exerted a detrimental effect on antitumor activity of other immunocytes. For example, Karyampudi et al. found that PD-1 was upregulated in tumor-infiltrating DC and mediated inhibition of NF-κB-dependent cytokine production, antigen presentation and costimulatory molecule expression [90]. Another recent study also showed that PD-1 expression on TAM negatively correlated with phagocytic potency against tumor cells, and blockade of PD-1/PD-L1 increased macrophage phagocytosis, reduced tumor growth, and lengthened survival in mice [91].

CTLA-4 is another inhibitory receptor expressed on activated T cells. As previously mentioned, the activation of T cells required costimulatory signals in conjunction with TCR signaling, among which, interaction between B7 on APCs and CD28 on T cells is most well-known. As a member of CD28 family, CTLA-4 shares the same ligand with CD28 but has a higher affinity with the ligand. Consequently, CTLA-4 competitively binds with B7 and leads to dysfunction of T cells. Moreover, during endocytosis of receptor, the ligand can be internalized together with CTLA-4 and degraded inside CTLA-4-expressing cells, leading to lack of costimulatory ligand for CD28 and thereby a raised threshold for T cell activation [92]. Besides, CTLA-4 is also constitutively expressed on Tregs and plays a critical role in Treg-mediated immunosuppression [93].

### Targeting microbiome in ICIs

Under normal conditions, the above-mentioned immune checkpoints are essential for preventing overstimulation of immune responses and maintaining immune tolerance to self-antigens. However, in the context of tumor, they are associated with compromised antitumor immunity and poor clinical outcomes. ICIs such as anti-CTLA-4 antibody and anti-PD-1/PD-L1 antibody, can specifically block these immune checkpoints and potentiate antitumor immunity, and is therefore regarded as a breakthrough in cancer immunotherapy. Its efficacy has been acknowledged in many malignancies such as melanoma, NSCLC and renal cell carcinoma (RCC). However, not all patients respond well to therapy. Potential biomarkers for therapeutic prediction include PD-L1 expression, tumor mutation burden, microsatellite instability-high and tumor-infiltrating lymphocytes, but none of which can fully explain the difference of therapeutic response. Recent data indicated a potential for gut microbiota in improving clinical response to ICIs [31–33, 36, 94]. As early as 2015, Vetizou et al. found that germ-free mice showed impaired antitumor effects of CTLA-4 blockade while recolonized germ-free mice with bacterial species such as *Bacteroides fragilis* could recover the therapeutic response [31]. Sivan et al. also showed that mice with different commensal microbes exhibited difference in melanoma growth rate and response to PD-L1 blockade, which could be eliminated by cohousing and fecal FMT [32]. Further analysis identified *Bifidobacterium* as a positive regulator of therapeutic response [32]. These pre-clinical models suggested that gut microbiota was required for the efficacy of ICIs. Gopalakrishnan et al. confirmed the hypothesis in cancer patients [33]. In the setting of anti-PD-1 treatment, they discovered significant differences in the diversity and composition of gut microbiome from responders versus non-responders [33]. A favorable gut microbiome, characterized by higher α diversity and a relative abundance of Ruminococcaceae, is associated with a better clinical outcome after anti-PD-1 therapy [33]. Similar result was discovered in Chinese patients [29]. Subsequently, more commensals were found to correlate with clinical benefit from ICIs (Table 1), such as *Akkermansia muciniphila* [94], *Bacteroides thetaiotamicron* [95], *Faecalibacterium* genus and other Firmicutes [41]. Recolonized germ-free mice with fecal samples from responders or dominant microbiota in responders could improve antitumor effects of ICIs [36, 94].

**Table 1:** Association between microbial taxonomic/metabolomic profiles and therapeutic response to ICI

Reference	Tumor (sample size)	Immunotherapy	Gut microbial taxonomic profiles	Gut microbial metabolomics profiles
Chaput et al. 2017 [41]	Metastatic melanoma (*n* = 26)	Ipilimumab	R had a baseline gut microbiome enriched with *Faecalibacterium* and other Firmicutes while NR showed a high abundance of *Bacteroides* at baseline. Patients whose baseline microbiota was enriched with *Faecalibacterium* genus and other Firmicutes, had longer PFS and OS than those with *Bacteroides* as dominant microbiota at baseline.	NA
Frankel et al. 2017 [95]	Metastatic melanoma (*n* = 39)	Ipilimumab (*n* = 1) or nivolumab (*n* = 1) or ipilimumab plus nivolumab (IN, *n* = 24), or pembrolizumab (P, *n* = 13)	R for all types of ICI therapies were enriched for *Bacteroides caccae*. Among R for IN, the gut microbiome was enriched with *Faecalibacterium prausnitzii*, *Bacteroides thetaiotamicron*, and *Holdemania filiformis*. R for P were enriched with *Dorea formicogenerans*. Overall gut microbiome diversity was not significantly different between R and NR.	R for all types of ICI therapies were enriched with bacterial enzymes involved in fatty acid synthesis. R for IN were enriched with bacterial enzymes involved in inositol phosphate metabolism.
Gopalakrishnan et al. 2018 [96]	Metastatic melanoma (*n* = 43)	Anti-PD-1	α diversity was significantly higher in R (*n* = 30) compared to NR (*n* = 13). Clostridiales/Ruminococcaceae/ *Faecalibacterium* was abundant in R while Bacteroidales was abundant in NR. A favorable gut microbiome, characterized by higher diversity and relative abundance of Ruminococcaceae, is associated with longer PFS.	Metagenomic WGS sequencing (*n* = 25) showed that anabolic functions including amino acid biosynthesis predominated in responders (*n* = 14), whereas catabolic functions predominated in NR (*n* = 11)
Matson et al. 2018 [36]	Metastatic melanoma (*n* = 42)	Anti-PD-1 or anti-CTLA-4	8 species were more abundant in R, including *Enterococcus faecium*, *Collinsella aerofaciens*, *Bifidobacterium adolescentis*, *Klebsiella pneumoniae*, *Veillonella parvula*, *Parabacteroides merdae*, *Lactobacillus spp.*, and *Bifidobacterium longum*, whereas *Ruminococcus obeum* and *Roseburia intestinalis* were more abundant in NR.	NA
Routy et al. 2018 [94]	NSCLC (*n* = 60) and RCC (*n* = 40)	Anti-PD-1	Species enriched in R included *Akkermansia muciniphila*, *Alistipes indistinctus*, *Enterococcus faecium* and some unclassified Firmicutes. An abundance of *Akkermansia muciniphila* in gut microbiota was associated with longer PFS.	NA
Zheng et al. 2019 [97]	HCC (*n* = 8)	Anti-PD-1	Over the entire treatment, R showed higher taxa richness and more gene counts than those of NR. 20 R-enriched species and 15 NR-enriched species were identified. In NR, proteobacteria markedly increased and replace bacteroidetes to become predominant at week 12. The dynamic-variation of the gut microbiome might be used for early prediction of the six-month outcomes of anti-PD-1 in HCC.	Functional analysis identified positive correlations between pathway (such as carbohydrate metabolism and methanogenesis), and R-enriched species.
JIN et al. 2019 [29]	Advanced NSCLC (*n* = 37)	Nivolumab	Fecal samples at baseline were obtained from 25 patients. R (*n* = 13) harbored higher diversity of baseline microbiome than NR (*n* = 12). High microbiome diversity is associated with prolonged PFS. No statistical difference at the phylum level was observed between R and NR at baseline or after baseline. At the genus level, *Alistipes putredinis*, *Bifidobacterium longum*, and P*revotella copri, Shigella Lachnobacterium,* and *Lachnospiraceae,* were enriched in R whereas *Ruminococcus unclassified* were enriched in NR.	NA
Peters et al. 2019 [98]	Metastatic melanoma (*n* = 27)	Anti-PD-1 or anti-CTLA-4 or anti-PD-1 combined with anti-CTLA-4	Higher microbial richness was associated with longer PFS. Abundance of *Bacteroides ovatus*, *Bacteroides dorei*, *Bacteroides massiliensis*, *Ruminococcus gnavus*, and *Blautia producta* were related to shorter PFS, and *Faecalibacterium prausnitzii*, *Coprococcus eutactus*, *Prevotella stercorea*, *Streptococcus sanguinis*, *Streptococcus anginosus*, and *Lachnospiraceae bacterium 3 1 46FAA* to longer PFS.	Pathway of L-rhamnose degradation, guanosine nucleotide biosynthesis, and B vitamin biosynthesis were related to shorter PFS.
Derosa et al. 2020 [99]	Advanced RCC (*n* = 69)	Nivolumab	Among no-ATB patients (*n* = 58), higher diversity of gut microbiome (both at the gene count and metagenomic species level) correlated with better response to nivolumab and longer PFS. *Akkermansia muciniphila*, *Bacteroides salyersiae*, and *Eubacterium siraeum* were enriched in R, whereas *Clostridium hathewayi*, *Clostridium clostridioforme* and *E. bacterium_2_2_44A* were enriched in NR.	NA
Li et al. 2020 [100]	Metastatic HCC (*n* = 65)	ICI	R showed a high abundance of Clostridiales/Ruminococcaceae in baseline fecal microbiome while NR has a high abundance of Bacteroidales. Patients with a high abundance of *Faecalibacterium* genus abundance had a significantly prolonged PFS versus those with a low abundance. Conversly, a high abundance of Bacteroidales was associated with a shortened PFS.	NA
Coutzac et al. 2020 [101]	Metastatic melanoma (*n* = 38)	Ipilimumab	Genera linked to long-term clinical benefit (PFS > 6 months) were *Faecalibacterium* and *Gemminger*. High relative abundance of *Faecalibacterium* at baseline was linked to OS over 18 months and longer PFS.	NA

*ICI* Immune checkpoint inhibitors, *R* Responders, *NR* Non-responders, *PFS* Progression-free survival, *OS* Overall survival, *NA* Non-applicable, *WGS* Whole genome shotgun, *NSCLC* Non-small-cell lung cancer, *RCC* Renal cell carcinoma, *HCC* Hepatocellular carcinoma, *ATB* Antibiotics

Of note, there are also specific bacteria whose abundance was correlated with insensitivity to immunotherapy (Table 1). Zheng et al. reported the dynamic variation of gut microbiome during anti-PD-1 immunotherapy in HCC patients [97]. In non-responders, proteobacteria markedly increased and became predominant at week 12 [97]. Chaput et al. found that high proportions of *Bacteroides* were present at baseline in patients with poor clinical benefit from ICI [41], which contradicted previous data showing the synergy of *Bacteroides* species in ICI [31, 95]. Low concordance between microbiota-related studies may be attributed to the techniques used for microbiome analysis, highlighting the importance of standardizing techniques for microbiome analysis [95]. It is believed that metagenomic shotgun sequencing (MSS) is superior to the more commonly used 16S RNA sequencing because it can not only avoid PCR bias derived from the choice of primers and 16S rRNA variable region, but also shed light on functional pathways [95, 102]. More importantly, MSS is better from the standpoint of higher resolution since bacteria belonging to the same genus can exhibit totally different effects on tumor immunity and immunotherapy [103]. In addition, host variables can make a difference to gut microbiome but most microbiota-targeted studies did not take these confounding factors into account. A recent study demonstrated host variables, such as alcohol intake frequency and bowel movement quality, could exert great influence on microbial composition [104]. Hence, when investigating the association between cancer/therapeutic response and gut microbiota, selecting comparison groups without adjusting these host factors may obtain spurious correlation. Matching cases and controls for confounding variables can reduce differences in the microbiota, and increase robustness and reproducibility in identifying bacterial taxa that are truly associated with human disease [104].

Since gut microbiota are associated with therapeutic response, any medications that can alter the gut microbiota may affect the efficacy of ICIs. Routy et al. investigated cancer patients for antibiotics usage within 2 months before, or 1 month after the first treatment of PD-1/PD-L1 inhibitor [94]. They found antibiotics compromised the clinical benefit, which manifested as shorter PFS and overall survival (OS) [94]. However, proton pump inhibitors (PPI) did not affect treatment efficacy, which may be attributed to the fact that PPI did not alter the diversity of gut commensals [35]. Interestingly, gut microbiota also correlated with the occurrence of immune-related adverse events (irAEs) induced by ICIs. Pretreatment with vancomycin induced a much earlier onset and more severe anti-CTLA-4-induced colitis in mice, whereas *Bifidobacterium* administration could ameliorate colitis without affecting the anticancer response [105]. Likewise, another study identified *B. breve* and *L. rhamnosum* as the two functional species responsible for alleviating intestinal irAEs [106]. A challenge to microbiota-targeted immunotherapy is how to balance efficacy and irAEs since ICI-mediated immune reactivation is not confined to TME. 16S rRNA sequencing showed that Firmicutes was dominant in baseline microbiota of patients prone to develop ICI-induced colitis, while Bacteroidetes phylum was associated with resistance to colitis [41]. These results were consistent with previous data showing that the presence of irAEs predicts a better clinical outcome in the context of ICIs [107–110]. However, 11 bacterial strains isolated from healthy donor feces could simultaneously enhance ICI sensitivity and attenuate their colitogenic side effects in recipient mice with adenocarcinoma [111]. More evidence is expected before we can properly manipulate gut microbiota to enhance efficacy as well as alleviate irAEs.

Although tremendous data have indicated that antibiotics administration adversely affects outcomes of ICIs (Table 2), potential biases make it difficult to include antibiotics into practice of cancer immunotherapy. Firstly, these studies are usually retrospective analysis without intervention. The class, dosage and duration of antibiotics which can make a great difference to the composition of microbiome cannot be unified. Moreover, patients treated with antibiotics may recover back to their original microbiome compostion before the first treatment of ICIs. Hence, it is essential to analyze microbiota composition after antibiotics usage and before treatments. Secondly, antibiotics are indicated for infection, which means the baseline inflammatory status in vivo of the antibiotics group differs from the control group. It is possible that infection, especially severe infection, has an adverse impact on efficacy of ICIs and prognosis. Thirdly, the functions of antibiotics are not restricted to disruption of commensals. It is increasingly appreciated that antibiotics play a complex role in cancer development and treatment. On one hand, some antibiotics are used as anticancer drugs via mechanisms independent from microbiome. These antibiotics are mainly peptides and anthraquinones, with anti-proliferative, pro-apoptotic and anti-EMT properties [134]. On the other hand, excessive administration of antibiotics can also result in cancer via intestinal dysbiosis-induced chronic inflammation, changes in normal tissue metabolism or direct genotoxicity [134, 135]. Therefore, reduced clinical benefit from ICIs cannot simply be attributed to the depletion of gut microbiota. At last, the sample size of antibiotics group is relatively small, which may lower the credibility of the conclusions.

**Table 2:** Recent studies investigating the association between antibiotics use and ICI efficacy in cancer patients

Tumor (sample size)	ICI	ATB Exposure	Results	Reference
NSCLC(*n* = 74)	Nivolumab	Those receiving ATB 3 months before the first nivolumab injection or during treatment	15 (20.3%) patients received ATB. ATB medication has no impact on either response rate to PD-1 blockade or PFS.	Kaderbhai et al. 2017 [112]
NSCLC (*n* = 74)	PD-1 inhibitors	Those receiving ATB within 6 weeks before initiation of PD-1 inhibitors	18 (24%) patients received ATB. ATB use did not impact ORR but was associated with worse OS and PFS even in multivariate analysis.	Thompson et al. 2017 [113]
Melanoma(*n* = 39)	Ipilimumab (*n* = 1), nivolumab (*n* = 1), ipilimumab plus nivolumab(*n* = 24), or pembrolizumab (*n* = 13)	Those receiving ATB or probiotics before or during ICI treatment.	3 (8%) patients received ATB and 1 (3%) received probiotics. Neither clinical response nor toxicity was associated with antibiotic or probiotic use.	Frankel et al. 2017 [95]
Advanced cancer(*n* = 60)	PD-1 inhibitors (nivolumab or pembrolizumab) or PD-L1 inhibitor (atezolizumab)	Those receiving ATB within 2 weeks prior to and after ICI initiation and within 10 weeks prior to disease progression.	17 (28%) patients received systemic antibiotics. They had a lower RR and shorter PFS. Multivariate analysis identified antibiotics as the only factor affecting RR and PFS. Patients who received broad-spectrum antibiotics experienced shorter OS.	Ahmed et al. 2018 [114]
NSCLC(*n* = 239), RCC(*n* = 121)	Patients with RCC received anti-PD-(L)1 mAb alone (*n* = 106) or in combination with anti-CTLA-4 mAb (*n* = 10) or bevacizumab (*n* = 5). Patients with NSCLC received anti-PD-(L)1 mAb alone (*n* = 205) or combined with anti-CTLA-4 mAb (*n* = 34)	Those receiving ATB within 30 days of beginning ICI	16 (13%) RCC patients and 48 (20%) NSCLC patients received ATB. In multivariate analyses, ATB was associated with shorter PFS in RCC and shorter OS in NSCLC.	Derosa et al. 2018 [115]
Non-squamous NSCLC (*n* = 30)	Nivolumab (*n* = 25) or pembrolizumab (*n* = 5)	Those receiving ATB within 1 month before and 1 month after ICI initiation.	11 (36.7%) patients received ATB. Median PFS and OS were significantly shorter in ATB group. In a multivariate analysis, ATB use was identified as the only parameter significantly associated with PFS and OS.	Huemer et al. 2018 [116]
NSCLC (*n* = 168)	Nivolumab (92.3%) or pembrolizumab (7.7%)	Those receiving ATB within 2 months before and 1 month after ICI initiation	47.9% patients received ATB. Patients who received ATB had shorter OS and PFS. The patients receiving ATB intravenously had a shorter OS and PFS than orally.	Mielgo-Rubio et al. 2018 [117]
NSCLC (*n* = 90)	Nivolumab	Those receiving ATB for ≥3 days within 30 days prior to nivolumab	13 (14.4%) patients received ATB. In multivariate analysis, no significant association was observed between survival and previous antibiotic use.	Hakozaki et al. 2019 [118]
Melanoma(*n* = 74)	Anti-PD-1 mAb alone (*n* = 54) or anti-CTLA-4 mAb alone (*n* = 5) or anti-CTLA-4 mAb plus chemotherapy (*n* = 15)	Those receiving ATB within 30 days before ICI initiation	10 (13.5%) patients received ATB. Patients who received ATB experienced more PD and shorter PFS.	Elkrief et al. 2019 [119]
NSCLC(*n* = 109)	Anti-PD-1 mAb alone (*n* = 57) or anti-PD-1 mAb plus chemotherapy (*n* = 33) or anti-PD-1 mAb plus anti-angiogenic agent (*n* = 19)	Those receiving ATB within 1 month before or after the first administration of PD-1 blockade	20 (18.3%) patients received ATB. In multivariable analysis, ATB treatment was markedly associated with worse PFS and OS.	Zhao et al. 2019 [120]
NSCLC(*n* = 119), melanoma(*n* = 38), other types(*n* = 39)	ICI	Those receiving ATB within 30 days prior to (pATB) or concurrent with (cATB) ICI therapy	pATB therapy, but not cATB therapy, was associated with worse OS and a higher likelihood of primary disease refractory to ICI therapy. Multivariate analyses confirmed the association between pATB therapy and OS.	Pinato et al. 2019 [121]
NSCLC (*n* = 37)	Nivolumab	Those receiving ATB within 2 months before clinical assessment	11 (29.7%) patients received ATB within 2 months. However, the R/NR ratio was similar in ATB and no-ATB groups.	Jin et al. 2019 [29]
Urothelial carcinoma (*n* = 101)	PD-1/PD-L1 inhibitors	Those receiving ATB within 1 months before or during ICI treatment	26 (25.7%) patients received ATB. Antibiotics compromised clinical outcomes significantly.	Agarwal et al. 2019 [122]
NSCLC (*n* = 157)	PD-1/PD-L1 inhibitor (*n* = 150) or CTLA-4 inhibitor (*n* = 1) or combination (*n* = 6)	1 months before or 3 months after ICI treatment was defined early immunotherapy period (EIOP). Antibiotic-immunotherapy exposure ratio (AIER) defined as “days of antibiotic/days of immunotherapy” during the whole immunotherapy period (WIOP) was also calculated.	46 (29.3%) patients received ATB during WIOP, 27 (17.2%) patients received ATB during EIOP. ATB use during EIOP has no impact on either PFS or OS. But the patients with a higher AIER had worse PFS and OS.	Galli et al. 2019 [123]
Esophagogastric cancer (*n* = 161)	Anti-PD-1/PD-L1 (*n* = 110) or anti-PD-1/PD-L1 combined with anti-CTLA-4 (*n* = 51)	Those receiving ATB within 2 months before or during ICI treatment	62 (38%) patients received ATB. No difference in PFS or OS between those patients treated with antibiotics versus those who were not.	Greally et al. 2019 [124]
Solid cancer (*n* = 234)	ICI alone or ICI combination or ICI combined with chemotherapy	Those receiving ATB within 60 days before ICI initiation	108 (46.2%) patients received ATB. ATB use was associated with a decreased OR, shorter PFS and OS. In the multivariate analysis, antibiotics use was a significant predictor of patient survival.	Kim et al. 2019 [125]
NSCLC (*n* = 72)	Nivolumab	Those receiving ATB within 2 months before and 1 month after ICI initiation	30 (42%) patients received ATB. ATB use was associated with shorter OS.	Krief et al. 2019 [126]
RCC (*n* = 146)	PD-1/PD-L1 inhibitors	Those receiving ATB within 8 weeks before and 4 weeks after ICI initiation	31 (21%) patients received ATB. ATB use was associated with a lower objective response rate and shorter PFS.	Lalani et al. 2020 [127]
NSCLC (*n* = 218)	Anti-PD-1 mAb alone (*n* = 207) or anti-PD-1 mAb plus chemotherapy (*n* = 5) or investigational immunotherapy^a^ (*n* = 6)	Those receiving ATB within 2 months before ICI treatment	33 (15.1%) patients received ATB. PFS and OS were significantly shorter in patients receiving ATB.	Schett et al. 2020 [128]
NSCLC(*n* = 1512)	Randomly assigned to receive atezolizumab (*n* = 757) or docetaxel (*n* = 755)	Those receiving ATB within 30 days before and 30 days after the first treatment	169 (22.3%) patients in the atezolizumab group received ATB. Multivariate analysis in all patients revealed that ATB were associated with shorter OS. Within the atezolizumab population, OS was significantly shorter in patients who received ATB.	Chalabi et al.2020 [129]
RCC(*n* = 69)	Nivolumab (3 mg/kg i.v. q2w)	Those receiving ATB use within 60 days of nivolumab	11 (16%) patients received ATB. Patients who received ATBs had a lower ORR, PFS and OS.	Derosa et al. 2020 [99]
NSCLC(*n* = 140), RCC(*n* = 55)	Single-agent ICI	Those receiving antibiotics within 4 weeks before and 6 weeks after the ICI initiation	54 (39%) NSCLC and 24 (44%) RCC patients received ATB. In multivariable analysis, PFS and OS were shorter in NSCLC patients who received broad-spectrum anti-anaerobes or ‘other’ antibiotics (vancomycin predominant). In RCC, patients who received penicillins /penicillin-class/early-generation cephalosporins had shorter PFS.	Kulkarni et al. 2020 [130]
Advanced cancer (*n* = 291, including 179 melanoma, 64 NSCLC and 48 RCC)	ICI	Those receiving ATB within 2 weeks before and 6 weeks after ICI initiation	92 (32%) patients received ATB. ATB use was associated with shorter PFS and OS in multivariate analysis. Administration of a single course of ATB had non-significant impact on PFS and OS while patients who received cumulative ATB for>7 days had significantly worse PFS and OS.	Tinsley et al. 2020 [131]
A meta-analysis included 19 eligible studies comprising 2740 cancer patients	Anti-PD-1/PD-L1 mAb (*n* = 14), anti-PD-1/PD-L1 mAb and/or anti-CTLA-4 mAb (*n* = 3), no information of the type of ICI drug (*n* = 2)	Pre-therapy ATB use (*n* = 11), post-therapy ATB use (*n* = 1), pre- or post-therapy ATB use (*n* = 9)	ATB use was negatively associated with OS and PFS in cancer patients. Similar results were obtained in the subgroup analyses stratified by the time of ATB use and cancer type.	Huang et al. 2019 [132]
A meta-analysis included 33 eligible studies comprising 5565 cancer patients	ICI (anti-PD−/PD-L1 or anti-CTLA-4) alone or combined with chemotherapy/targeted therapy.	ATB use prior to or within therapy	ATB use was significantly correlated with worse OS and PFS. The similar results were also found in subgroup analysis for lung cancer (both OS and PFS), RCC (only significant in PFS) and other cancers. The ICI efficacy was more likely to be diminished by ATB administration within a time frame from 60 days before to 60 days after ICI initiation.	Yang et al. 2020 [133]

*ICI* Immune checkpoint inhibitor, *ATB* Antibiotics, *pATB* ATB therapy administered prior to ICI, *cATB* ATB therapy administered concurrently, *NSCLC* Non-small-cell lung cancer, *RCC* Renal cell carcinoma, *PD* Progressive disease, *PFS* Progression-free survival, *OS* Overall survival, *ORR* Overall response rate, *mAb* Monoclonal antibody, *A. muciniphila Akkermansia muciniphila*, *B. salyersiae Bacteroides salyersiae*, *FMT* Fecal material transfer

^a^ Six patients received investigational immunotherapy (five patients received the ICI PDR-001 in combination with the oral adenosin receptor antagonist NIR-178, one patient received nivolumab in combination with a F16-IL2 fusion protein)

In addition to antibiotics, other strategies to modulate gut microbiome are also promising in cancer immunotherapy. A recent single-arm study evaluated the safety and efficacy of responder-derived FMT combined with anti-PD-1 in anti-PD-1-resistant melanoma [136]. This combination was well-tolerated and reversed the insensitivity to PD-1 blockade in 6 of 15 patients [136]. Similarly, another phase I clinical trial observed clinical responses in 3 of 10 anti-PD-1 refractory melanoma patients after FMT [137]. Both studies demonstrated that FMT can change the gut microbiome, which reprogrammed the TME to overcome resistance to ICI. The therapeutic value of microbiota modulation in cancer immunotherapy remains to be proven in more well-designed clinical trials enrolling larger sample sizes (Table 3).

**Table 3:** Ongoing clinical trials investigating the association between gut microbiome interventions and immunotherapy

Tumor (estimated enrollment)	Intervention (intervention model)	Primary outcome	Secondary outcome	status	ClinicalTrials.gov Identifier
Advanced RCC (*N* = 30)	*Clostridium butyricum* probiotic strain (CBM588) in combination with nivolumab/ipilimumab (parallel assignment)	Change in Bifidobacterium composition of stool	Change in Shannon index; ORR; PFS	Recruiting	NCT03829111
Solid tumor (*N* = 132)	Probiotic strain (MRx0518^a^) in combination with pembrolizumab (single group assignment)	Safety and clinical benefit of MRx0518 in combination with pembrolizumab	ORR; DoR; DCR; PFS	Recruiting	NCT03637803
Operable stage I-III breast cancer	Probiotics RBX7455 prior to surgery (single group assignment)	Safety	Systemic immunomodulatory effects	Recruiting	NCT04139993
Advanced melanoma (actual enrollment = 14)	Experimental: vancomycin pretreatment plus oral microbiome intervention (SER-401) in combination with nivolumab (parallel assignment)	Percentage of patients with AEs	ORR; DCR; PFS; OS; DoR; Change in the percentage of CD8^+^cells in tumor tissue	Active, not recruiting	NCT03817125
Surgically resectable pancreatic cancer (actual enrollment = 0)	Antibiotics in combination with pembrolizumab (single group assignment)	Change in immune activation in pancreatic tumor tissue	NA	Withdrawn (suspended due to primary investigator’s decision)	NCT03891979
Advanced lung adenocarcinoma (*N* = 30)	Oral RMT capsule in combination with durvalumab/durvalumab plus chemotherapy (single group assignment)	ORR; Safety of RMT	PFS; OS; DoR; irAEs; ORR; QoL;	Not yet recruiting	NCT04105270
Solid tumor (*N* = 65)	MET^b^ (MET-4 strains) in combination with ICIs (parallel assignment)	Cumulative relative abundance of ICI-responsiveness associated species; Changes in relative abundance of ICI-responsiveness associated MET-4 strains; Cases of treatment-related AEs	Cumulative relative abundance of ICI-responsiveness associated species at later time; Changes in relative abundance of ICI-responsiveness associated MET-4 strains at later time; Bacterial taxonomic diversity	Recruiting	NCT03686202
Castration-resistant metastatic prostate cancer (*N* = 32)	Responder-derived FMT in combination with pembrolizumab and enzalutamide (single group assignment)	Anticancer effect (Percentage of participants with a PSA decline of ≥50%)	Percent PSA change; Radiographic RR; Time to PSA progression; Time to radiographic progression; PFS; OS; Time to next therapy; Safety	Recruiting	NCT04116775
Anti-PD-1-resistant melanoma (*N* = 40)	Responder-derived FMT (single group assignment)	Incidence of FMT-related AEs; Changes in gut bacterial composition	Changes in composition and activity of immune cells	Recruiting	NCT03353402
Anti-PD-1-resistant advanced melanoma (*N* = 20)	Responder-derived FMT in combination with pembrolizumab (single group assignment)	ORR	Change in T-cells composition, innate/adaptive immune system subsets and function of T-cells	Recruiting	NCT03341143
Advanced melanoma or NSCLC (*N* = 50)	Responder-derived FMT in combination with nivolumab (single group assignment)	Incidence of FMT-related AEs; ORR	Changes in immune activation in the gut and tumor; PFS; OS; DoR; Anti-PD-1-related immune toxicities	Not yet recruiting	NCT04521075
Advanced melanoma (*N* = 20)	A healthy donor-derived FMT in combination with pembrolizumab/nivolumab (single group assignment)	Safety of combining FMT and immunotherapy	ORR; changes in gut microbiome, immune blood biomarkers and metabolomics	Recruiting	NCT03772899
Anti-PD-1-resistant gastrointestinal cancers (*N* = 10)	FMT in combination with anti-PD-1 (single group assignment; healthy people who have the gut microbiota profile similar to the responders of anti-PD-1 will be identified as donor)	ORR; AEs; Rate of abnormal vital signs and laboratory test results	Change in T-cells composition, subsets of immune system; Function of T-cells; Association of anti-PD-1 response with gut microbiota; AEs; Rate of abnormal vital signs, PE and ECG and laboratory test results	Recruiting	NCT04130763
Metastatic colorectal adenocarcinoma (*N* = 15)	Responder-derived FMT in combination with pembrolizumab /nivolumab (single group assignment)	ORR	NA	Not yet recruiting	NCT04729322
RCC (*N* = 20)	Healthy donors-derived FMT in combination with nivolumab and ipilimumab (single group assignment)	Occurrence of immune-related colitis	Incidence of irAEs; ORR; Change in microbiome and immune response; QoL	Recruiting	NCT04163289
Postoperative stage II/III CRC (*N* = 294)	Oral metronidazole before postoperative chemotherapy (parallel assignment)	DFS	OS; RR	Recruiting	NCT04264676

*DCR* Disease control rate, *AEs* Adverse events, *DoR* Duration of response, *RMT* Oral restorative microbiota therapy, *QoL* Quality of life, *MET* Microbial ecosystem therapeutics, *RMT* Restorative microbiota therapy

^a^ MRx0518 is a live biotherapeutic product consisting of a lyophilised formulation of a proprietary strain of bacterium

^b^ Microbial Ecosystem Therapeutics (MET) is a new treatment approach developed as an alternative to FMT. MET consists of a defined mixture of pure live cultures of intestinal bacteria isolated from a stool sample of a healthy donor

## Conclusion and outlook

Accumulating evidence showed that commensal microbiota can influence antitumor immunity via various mechanisms. However, gut-microbiota-targeted immunotherapy still has a long way to go. Firstly, the relationship between the gut microbiome and cancer is multi-faceted and most likely bidirectional. It is important to clarify which genus or even species can be utilized to promote anti-tumor response in humans. Secondly, since most studies were done in mice or in vitro, more clinical studies are needed before extending the conclusion from mouse to human. In clinical practice, the situation is more complicated, since different tumor types and staging, previous treatments and various host factors can disrupt the composition of gut microbiota. Thirdly, the phenotypes and functions of immunocytes in TME were heterogeneous. Moreover, TME tends to induce an anergic or immunosuppressive phenotype, leading to therapeutic resistance. Hence, increasing tumor-infiltrating immunocytes is necessary but not sufficient for triggering an effective antitumor immune response. It is equally important to induce their polarization towards desired phenotype. ICIs, in combination with other therapies aimed to reverse the suppressive TME probably make a difference. Finally, some data showed that microbiota could only play an adjuvant role in the presence of other cancer therapy [27, 84], whereas some studies found that gut commensals were able to impact tumor growth independently [33, 138]. Whether some species deemed “useless” in previous studies can exert a role when combined with other conventional therapies has yet to be determined.

Altogether, existing evidence is only the “tip of the iceberg” of an elusive network between microbiome and tumor surveillance. Despite emerging data confirmed the potential of microbiota manipulation in improving clinical outcomes, a clearer understanding of the mechanisms underlying this interaction is needed before gut microbiota can be introduced into clinical practice as an adjuvant regimen, which is also the challenge in current and future work.

## Data Availability

Not applicable.

## Abbreviations

ADCC: Antibody-dependent cell-mediated cytotoxicity
APC: Antigen-presenting cells
CNS: Conserved non-coding sequence
COX2: Cyclooxyganese2
CRC: Colorectal cancer
CTL: Cytotoxic T lymphocytes
CTLA-4: Cytotoxic T-lymphocyte antigen-4
CTSK: Cathepsin K
CTX: Cyclophosphamide
DC: Dendritic cells
DFS: Disease-free survival
EMT: Epithelial mesenchymal transition
FMT: Fecal microbiota transplantation
FXR: Farnesoid X receptor
γδT17: IL-17-producing γδT cells
GPR: G protein-coupled receptor
HCC: Hepatocellular carcinoma
HDAC: Histone deacetylase
HSC: Hepatic stellate cells
ICAM-1: Intercellular adhesion molecule 1
ICI: Immune checkpoint inhibitors
ILC: Innate lymphoid cells
IMC: Immature myeloid cells
irAEs: Immune-related adverse events
LPS: Lipopolysaccharide
LTA: Lipoteichoic acid
MDSC: Myeloid-derived suppressor cells
MSS: Metagenomic shotgun sequencing
NASH: Non-alcoholic steatohepatitis
NF-κB: Nuclear factor-κB
NK: Natural killer cells
NKT: Natural killer T cells
NSCLC: Non-small-cell lung cancer
OS: Overall survival
PAMP: Pathogen-associated molecule pattern
PD-1: Programmed death 1
PD-L1: Programmed death ligand 1
PFS: Progression-free survival
pMHC: Peptide-major histocompatibility complex
PPI: Proton pump inhibitors
PRR: Pattern recognition receptor
RCC: Renal cell carcinoma
ROS: Reactive oxygen species
SCFA: Short-chain fatty acids
TAM: Tumor-associated macrophages
TCR: T cell receptor
Th: Helper T cells
TIL: Tumor-infiltrating lymphocytes
TLR: Toll-like receptor
TME: Tumor microenvironment
Treg: Regular T cells
